# The David Operation Offers Shorter Hemostasis Time Than the Bentall in Case of Acute Aortic Dissection Type A

**DOI:** 10.7759/cureus.21747

**Published:** 2022-01-30

**Authors:** Ryohei Ushioda, Tomonori Shirasaka, Taro Kanamori, Atsuko Fujii, Makoto Shirakawa, Taro Takeuchi, Hiroyuki Kamiya

**Affiliations:** 1 Cardiac Surgery, Asahikawa Medical University, Asahikawa, JPN; 2 Cardiovascular Surgery, Kawaguchi Cardiovascular and Respiratory Hospital, Saitama, JPN

**Keywords:** hemostasis, aortic root replacement, david, bentall procedure, type a acute aortic dissection

## Abstract

Background

The aim of the present study was to compare the clinical outcome of the David operation and the Bentall operation in patients with Stanford type A acute aortic dissection (AADA) from the viewpoint of hemostasis.

Methods

Between April 2016 and April 2020, 235 patients underwent emergent surgery for AADA. Of them, 38 patients required aortic root replacement (ARR: The David operation 17, the Bentall operation 21). The mean age was 59.3±12.6 years. In the present series, the David operation was the first choice for relatively young people, and the Bentall operation was performed for relatively elderly patients and cases in which valve-sparing seemed impossible.

Results

Between the David and the Bentall group, the 30-day mortality rate did not differ significantly. However, hemostasis time (144.6±50.3 vs. 212.5±138.1 min, p=0.047), defined as the interval from the cessation of cardio-pulmonary bypass (CPB) to the end of the operation, and total operation time (477.8±85.7 vs. 578.3±173.6 min, p=0.027) were significantly shorter in the David group than in the Bentall group, and the amount of blood transfusion was less in the David group than in the Bentall group (red blood cells: 3.5±3.6 vs. 9.2±5.9 units, p=0.013; fresh frozen plasma: 4.1±4.7 vs 9.4±5.1 units, p=0.002; platelet concentrate: 33.2±11.3 vs 42.2±12.0 units, p=0.025).

Conclusion

David operation offers a shorter hemostasis time and consequently shorter operation time than the Bentall operation in the setting of AADA, probably due to double suture lines, despite its surgical complexity.

## Introduction

Some patients with acute aortic dissection type A (AADA) require aortic root replacement (ARR) because of the enlarged aortic root, extensive tissue destruction of the aortic root, and/or intimal tears located in the aortic root. In such patients, the Bentall operation using a composite graft with a vascular prosthesis and an artificial valve used to be the gold standard [1-3], but in recent years, valve-sparing ARR (VSRR) has been gaining attention, especially for young patients [4-7]. However, VSRR is a more complex and time-consuming procedure than the Bentall operation, and, therefore VSRR, has not been widely adopted in patients with AADA despite its potential benefits.

There are two modalities of VSRR, i.e., the re-implantation technique by David [8] and the remodeling technique by Yacoub [9]. Of them, the David operation requires two suture lines: first-row stitches from the left ventricular outflow tract to the outside of the aortic root for anchoring the vascular prosthesis, and second-row stitches to attach the aortic wall of the sinus of Valsalva to the inside of the vascular prosthesis [8]. Because of the two suture lines, the David operation may provide better hemostasis than the Bentall operation, especially in patients with AADA, because coagulopathy and very fragile tissue are very common in AADA patients. The aim of the present study was to compare the David operation and the Bentall operation in patients with AADA from the viewpoint of hemostasis.

## Materials and methods

The study protocol was reviewed and approved by our institutional review board, and the need for additional informed consent was waived (IRB number: 20200808. Name of IRB name: Kawaguchi Cardiovascular and Respiratory Hospital Institutional Review Committee).

Between April 2016 and April 2020, 235 patients underwent emergency surgery for AADA. Of them, 38 patients who needed ARR were retrospectively evaluated in the present study; their mean age was 59.3±12.6 years, and 31 (81.6%) were male. The David operation was performed in 17 patients, and the Bentall operation was performed in 21 patients (biological valve in 14 patients and mechanical valve in seven patients). The standard indications for ARR in the setting of AADA are extensive tissue destruction, the presence of a concomitant aortic root aneurysm ≥4.5 cm, or a known connective tissue disorder. In the present series, the David operation was the first choice for young people, and the Bentall operation was performed for elderly patients and cases in which self-valve-sparing seemed impossible.

All patients were intubated under general anesthesia, and they were maintained on mechanical ventilation during the operation. A standard median sternotomy was performed, and cardiopulmonary bypass (CPB) was instituted. The arterial cannula was inserted into the femoral artery or into the ascending aorta using the Seldinger technique. The venous cannula was generally inserted through the right atrial appendage, but selective bi-caval venous cannulation was performed in some cases. A venting cannula was inserted through the right superior pulmonary vein to the left ventricle. The ascending aorta was clamped if there was no thick thrombus in the false lumen. Otherwise, the ascending aorta was not clamped, and myocardial protection was achieved at the time of the hypothermic circulatory arrest. Myocardial protection was achieved by intermittent antegrade and/or retrograde administration of hypothermic blood cardioplegia. The hypothermic circulatory arrest was induced at a rectal temperature of 25 °C. All three arch vessels were cannulated, and antegrade selective cerebral perfusion was started. Entry resection was the standard procedure in our institute, and partial arch replacement with the reconstruction of the brachiocephalic trunk with or without reconstruction of the left common carotid artery, or total arch replacement was done according to the location of the entry. Technical details of the David operation and the Bentall operation have been described elsewhere. The Carrel patch was the first choice for coronary reconstruction, but the Cabrol method or coronary artery bypass grafting was added if needed.

Follow-up

Follow-up information on all patients was collected through planned outpatient visits in the course of regular clinical follow-up. The follow-up rate was 100%, and the mean duration of follow-up was 435±433 days.

Statistical analysis

Results are expressed as means±standard deviation. Statistical analysis was performed using Student’s t-test for continuous variables or chi-square tests (Fisher’s exact tests if n<5) for categorical variables. Kaplan-Meier analysis was used to compare late mortality between groups. A P-value less than 0.05 was considered significant. All statistical analyses were performed using SPSS 22.0 software (SPSS Inc., Chicago, IL, USA).

## Results

The average age of the patients was 51.4±8.6 years in the David group and 65.7±12.1 years in the Bentall group (p<0.001), and more than 80% of them were male. Generally, the Bentall group had a more critical preoperative condition than the David group, such as malperfusion (2/17 in the David group vs 12/21 in the Bentall group; p=0.004) and cardiac tamponade with shock (2/17 in the David group vs 9/21 in the Bentall group; p=0.018). Malperfusion of organs in the Bentall group was frequently presented in the cerebrum (n=5, 23.8%) and coronary (n=4, 19.1%). Moreover, preoperative blood tests showed lower platelet (19.28±11.28x10^4/µL in the David group and 16.43±5.38x10^4/µL in the Bentall group; p=0.0023), and extended APTT (35.18±6.97 sec in the David group and 37.45±23.1 sec in the Bentall group; p=0.0001) in the Bentall group. The value of other coagulation factors, serum acidosis, and serum lactate was similar between the groups. As for the indications for ARR, the David group had more root enlargement (12/17 in the David group vs 5/21 in the Bentall group; p=0.002), but the rate of rupture of the aortic root and entry in the aortic root was similar in the two groups. The demographic characteristics of the groups are summarized in Table 1.

**Table 1:** Patients’ characteristics and preoperative data COPD - Chronic obstructive pulmonary disease; PLT - platelet; PT-INR - prothrombin time - international normalized ratio; APTT - activated partial thromboplastin time; FIB - fibrinogen

Variable	David (n=17)	Bentall (n=21)	P-value
Age (y)	51.4±8.6	65.7±12.1	0.0001
Male (n)	14 (82.3%)	17 (80.9%)	0.456
Preexisting comorbidities			
Hypertension (n)	6 (35.3%)	5 (23.8%)	0.219
COPD (n)	0 (0%)	2 (9.5%)	0.096
Diabetes mellitus (n)	1 (5.9%)	0 (0%)	0.130
Chronic renal disease (n)	1 (5.9%)	2 (9.5%)	0.340
Hyperlipidemia (n)	2 (11.7%)	2 (9.5%)	0.412
Current smoker (n)	2 (11.7%)	5 (23.8%)	0.170
Malperfusion (n)	2 (11.7%)	12 (57.1%)	0.004
Brain (n)	0 (0%)	5 (23.8%)	0.015
Coronary (n)	1 (5.9%)	4 (19.1%)	0.116
Mesentery (n)	1 (5.9%)	0 (0%)	0.130
Renal (n)	0 (0%)	1 (4.7%)	0.181
Extremities (n)	0 (0%)	1 (4.7%)	0.181
Spinal cord (n)	0 (0%)	2 (9.5%)	0.096
Preoperative Blood Tests			
PLT (x10^4/µL)	19.28±11.28	16.43±5.38	0.0023
PT-INR	1.16±0.25	1.29±0.36	0.215
APTT (sec)	35.18±6.97	37.45±23.1	0.0001
FIB (mg/dL)	196.71±115.38	163.47±88.8	0.319
pH	7.34±0.14	7.32±0.11	0.239
Lactate (mmol/L)	3.49±4.40	3.97±3.57	0.373
Preoperative condition			
Cardiac arrest (n)	1 (5.9%)	2 (9.5%)	0.340
Cardiac tamponade with shock (n)	2 (11.7%)	9 (42.8%)	0.018
Indication for root replacement			
Aortic root rupture (n)	4 (23.5%)	6 (28.6%)	0.363
Entry in the aortic root (n)	8 (47.1%)	9 (42.8%)	0.398
Root enlargement (n)	12 (70.5%)	5 (23.8%)	0.002

Operative data are shown in Table 2. The extent of arch replacement and concomitant procedures were not different between the groups. Therefore, CPB time, myocardial ischemia time, and hypothermic circulatory arrest time did not differ between the groups. However, hemostasis time (144.6±50.3 min in the David group and 212.5±138.1 min in the Bentall group; p=0.047), is defined as the interval from the cessation of CPB to the end of the operation, and total operation time (477.8±85.7 min in the David group and 578.3±173.6 min in the Bentall group; p=0.027) were significantly shorter in the David group than in the Bentall group. Furthermore, the amount of blood transfusion was less in the David group than in the Bentall group (red blood cells: 3.5±3.6 units in the David group vs 9.2±5.9 units in the Bentall group, p=0.013; fresh frozen plasma: 4.1±4.7 units in the David group vs 9.4±5.1 units in the Bentall group, p=0.002; platelet concentrate: 33.2±11.3 units in the David group vs 42.2±12.0 units in the Bentall group, p=0.025), although the bleeding amount did not differ between the groups.

**Table 2:** Operative data CABG - coronary artery bypass grafting; MVR - mitral valve replacement; CPB - cardiopulmonary bypass; HCA - hypothermic circulatory arrest; RBC - red blood cell; FFP - fresh frozen plasma; PC - packed cell

Variable	David (n=17)	Bentall (n=21)	P-value
Location of primary entry			
Root (n)	8 (47.1%)	9 (42.8%)	0.398
Ascending (n)	6 (35.3%)	7 (33.3%)	0.216
Arch (n)	4 (23.5%)	6 (28.6%)	0.363
Proximal descending	2 (11.7%)	2 (9.5%)	0.412
Extent of replacement			
Ascending	6 (33.3%)	9 (42.8%)	0.318
Partial arch	6 (35.3%)	7 (33.3%)	0.450
Total arch	5 (29.4%)	5 (23.8%)	0.348
Concomitant procedures			
CABG	2 (11.7%)	5 (23.8%)	0.171
MVR	0 (0%)	1 (4.8%)	0.181
Femoro-femoral bypass	0 (0%)	1 (4.8%)	0.181
Operation time (min)	477.8±85.7	578.3±173.6	0.027
CPB time (min)	297.4±56.8	336.1±113.2	0.182
Myocardial ischemia time (min)	248.7±61.6	257.9±63.6	0.655
HCA time (min)	48.6±21.2	45.3±16.4	0.603
Hemostasis time (min)	144.6±50.3	212.5±138.1	0.047
Bleeding amount (mL)	1253±545	1437±684	0.365
Blood transfusion			
RBC (units)	3.5±3.6	9.2±5.9	0.013
FFP (units)	4.1±4.7	9.4±5.1	0.002
PC (units)	33.2±11.3	42.2±12.0	0.025

Short-term outcomes are shown in Table 3. Reflecting the fact that patients in the David group were less severely ill than patients in the Bentall group, ICU stays (5.1±2.2 days in the David group and 7.3±5.7 days in the Bentall group; p=0.0001) and hospital stay (23.1±10.4 days in the David group and 38.0±27.2 days in the Bentall group; p=0.039) were significantly shorter in the David group than in the Bentall group. Regarding postoperative complications, no patient underwent re-thoracotomy for bleeding. Postoperative stroke (0/17 in the David group vs 5/21 in the Bentall group; p=0.015) and sepsis (0/17 in the David group vs 4/21 in the Bentall group; p=0.028) occurred significantly less in the David group than in the Bentall group, but there were no differences in other complications between the groups. Regarding 30-day mortality, two patients in the David group (one patient due to low output syndrome and the other patient due to acute subdural hematoma) and four patients in the Bentall group (one patient due to low output syndrome, one patient due to stroke, and the other two patients due to sepsis) died, but the 30-day mortality rate did not differ significantly between the groups. Regarding in-hospital mortality, one additional patient in the Bentall group died 45 days after the operation due to sepsis.

**Table 3:** Postoperative outcomes

Variable	David (n=17)	Bentall (n=21)	P-value
ICU stay (days)	5.1±2.2	7.3±5.7	0.0001
Hospital stay (days)	23.1±10.4	38.0±27.2	0.039
Complications			
Re-thoracotomy for bleeding (n)	0 (0%)	0 (0%)	1
Moderate AR (n)	1 (5.9%)	0 (0%)	0.130
Acute kidney injury requiring dialysis (n)	0 (0%)	1 (4.8%)	0.181
Pericardial effusion requiring drainage (n)	1 (5.9%)	4 (19.0%)	0.116
Respiratory failure requiring tracheotomy (n)	0 (0%)	1 (33.3%)	0.181
Stroke (n)	0 (0%)	5 (23.8%)	0.015
Paraplegia (n)	0 (0%)	2 (9.5%)	0.095
GI bleeding (n)	0 (0%)	2 (9.5%)	0.095
Sepsis (n)	0 (0%)	4 (19.0%)	0.028
30-day mortality (n)	2 (11.7%)	4 (19.0%)	0.540
In-hospital mortality (n)	2 (11.7%)	5 (23.8%)	0.341

**Figure 1:**
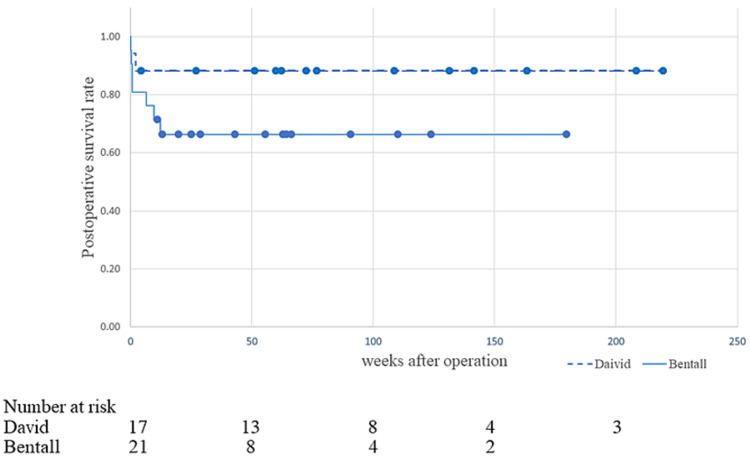
Survival curve in the David group and Bentall group

During the follow-up period, two patients in the David group (both within 30 days) and seven patients in the Bentall group (five within hospital stay, one due to colon perforation, and the other due to unknown cause) died. The survival curves are shown in Figure 1. Reflecting the fact that the Bentall group was more severely ill than the David group, the initial decrease in the survival curve was more obvious in the Bentall group, but there was no significant difference in late survival between the groups (p=0.156).

## Discussion

The crucial findings of the present study were: (1) the David operation can be performed with acceptable results in selected patients having AADA and (2) despite its surgical complexity, the David operation offers shorter hemostasis time and consequently shorter operation time than the Bentall operation in the setting of AADA, probably due to double suture lines.

The surgical approach for AADA differs between western countries and Japan. In general, aortic root surgery is more common in western countries, and aortic arch surgery is more common in Japan. Regarding aortic root surgery in patients with AADA, the frequency of root replacement was about 30% in Europe and the United States [10], whereas it is obviously low, at about 7%, in Japan [11]. On the other hand, the frequency of total arch replacement was about 15% in Europe and the United States and about 56% in Japan [10,11]. Our study's frequency of total arch replacement is less than 30% in both groups. The basic therapeutic strategy for acute aortic dissection is life-saving. We basically have an aggressive attitude to attack this etiology in the young adult, however, simultaneous operation of the aortic root and aortic arch in the acute aortic dissection cases is often involved with considerable cardiac arrested and CPB time, which may aggravate the bleeding tendency brought by malignant coagulopathy. Therefore, we put more priority on the exclusion of entry and repair of the aortic root in such situations and does not always attack the aortic arch.

In the present series, about 16% of patients with AADA underwent root replacement, and the rate is obviously higher than the Japanese average, but almost half of the average of Europe and the United States. However, among those patients who underwent ARR, about 60% underwent total arch replacement or partial arch replacement with a reconstruction of at least one arch vessel, and the rate was similar to the Japanese average, but almost four times higher than the average of Europe and the United States. In such an aggressive strategy, hemostasis, i.e., control of surgical bleeding and non-surgical coagulopathy, is very important.

However, it is sometimes difficult to distinguish surgical bleeding due to technical issues from stitch-hole bleeding due to coagulopathy, especially in patients with AADA, because many patients have coagulopathy preoperatively, and hypothermic circulatory arrest worsens this coagulopathy [12,13]. In the present series, due to the heterogeneity of concomitant surgical procedures, myocardial ischemia time, CPB time, and HCA time did not differ between the David group and the Bentall group, although the David operation is more time-consuming than the Bentall operation [14,15]. However, hemostasis time was defined from cessation of CPB to the end of the operation and total operation time was significantly shorter in the David group than in the Bentall group. These findings imply that the David operation is superior to the Bentall operation for local hemostasis at the aortic root in patients with AADA. There has been no similar study of AADA so far, but as mentioned above, the double suture line of the David operation is thought to reflect the ease of hemostasis at the base in AADA patients with coagulation abnormalities in the background. To the best of our knowledge, this is the first description of the effectiveness of the David operation from the viewpoint of hemostasis.

Despite its surgical complexity, many previous studies have suggested the non-inferiority of the David operation to the Bentall operation in patients with AADA [14-16]. For example, Yang et al. compared surgical outcomes in patients who underwent the David operation (n=40) and the Bentall operation (n=95) for AADA. Postoperative mortality was 18% in the entire cohort (David group 5% and Bentall group 23%). Ten-year Kaplan-Meier survival was 66% for the entire cohort, with 98% survival in the David group. They concluded that both the David operation and the Bentall operation are appropriate surgical approaches for ARR in select patients with an acute type A aortic dissection [16]. In the present study, ICU stay (p=0.0001) and hospital stay (p=0.039) were significantly shorter in the David group. In addition, there were no significant differences between the groups in 30-day mortality (David group 11.7% and Bentall group 19.0%) and in-hospital mortality (David group 11.7% and Bentall group 33.3%). According to data from the International Registry of Aortic Dissection, 30-day mortality in AADA ranges from 17% to 26% [10]. Similar to the above results, in the present series, the David operation did not compromise survival when compared with the Bentall operation, and it was comparable to other recent studies in which the David operation was used for AADA treatment [14-16].

Limitations

There are several limitations to the present study. First, this was a retrospective study of a single center's experience with a relatively small sample size. Moreover, the two groups in the present study had different patient backgrounds, with younger patients in the David group (p<0.001), and more severe cases, e.g., malperfusion (p=0.004) and cardiac tamponade with shock (p=0.018), low platelet (p=0.0023), extended APTT (p=0.0001) in the Bentall group. These factors are said to be higher the perioperative mortality [17-19]. Therefore, it may not be possible to simply compare the bleeding amount between the two groups. However, we still think it quite valuable to show our findings that the David operation can be performed with acceptable results in selected patients having AADA and despite its surgical complexity, the David operation offers shorter hemostasis time and consequently shorter operation time than the Bentall operation in the setting of AADA, probably due to double suture lines. In addition, because preoperative or intraoperative root dimensions were not recorded, surgical procedure selection was often based on the surgeon’s experience, impairing comparability and generalizability.

## Conclusions

The present study demonstrated that the David operation offers a shorter hemostasis time and consequently a shorter operation time than the Bentall operation in the setting of AADA, probably due to double suture lines, despite its surgical complexity. Therefore, especially for younger patients, this valve-sparing fashion seems valuable in terms of clinical benefits.
